# Nonabsorbable-Suture-Induced Osteomyelitis: A Case Report and Review of the Literature

**DOI:** 10.1155/2012/381490

**Published:** 2012-10-09

**Authors:** Cheng Hong Yeo, Nick C. Russell, Tom Sharpe

**Affiliations:** ^1^The Orthopaedics Department, Toowoomba Base Hospital The Queen Elizabeth Hospital, Perchey Street, Toowoomba QLD 4350, Australia; ^2^The Queen Elizabeth Hospital, 28 Woodville Road, Woodville West SA 5011, Australia

## Abstract

We are reporting a case of nonabsorbable suture-induced osteomyelitis in patient who had an open rotator cuff repair with nonabsorbable Ethibond anchor suture. Patient in this case presented with very subtle clinical features of osteomyelitis of the left proximal humerus 15 years after initial rotator cuff repair surgery. Literature had shown that deep infection following rotator cuff repairs, although rare, can be easily missed and can cause severe complications. Absorbable suture had been demonstrated to be more superior, in terms of rate of deep infection, as compared to nonabsorbable suture when used in rotator cuff repair surgery. Both absorbable and nonabsorbable suture had been demonstrated to have similar mechanical properties by several different studies. The case demonstrated that initial presentation of deep infection can be subtle and easily missed by clinicians and leads to further complications.

We present a case of a 71-year-old male, with known background history of ischaemic heart disease and chronic obstructive pulmonary disease, who presented to hospital with insidious onset left shoulder pain, 15 years after open rotator cuff repair with Ethibond suture. No systemic symptoms were elicited. On examination he was afebrile, with reduced range of motion secondary to pain. Investigations found C-reactive protein (CRP) of 113 mg/L and a white cell count (WCC) of 7.9 × 10^9^/L. X-ray showed an arthritic glenohumeral joint (Figure 1), and ultrasound showed no effusion. 

Subsequently his pain increased, and one week later his CRP had increased to 144 mg/L. He was taken to theatre for an arthroscopic washout for presumed septic arthritis, but findings were nonspecific. Biopsy specimens grew no bacteria on culture. During hospitalisation for further observations, his pain did not settle, and he was treated with empiric IV cefazolin. CT scan and MRI (Figure 2) showed signs of osteomyelitis of the proximal humerus. He returned to theatre for an open washout and debridement. Intraoperatively, a cortical defect on the bone was identified and chronic granulation tissue was cleared. A cyst (measuring 2.1 × 7.7 cm on MRI) was noted in the humeral head which contained the old Ethibond suture (Figure 3). Collection of joint fluid and soft tissue specimens that were obtained intraoperatively grew propionibacterium and histology showed chronic osteomyelitis. His antibiotic regime was changed to IV Tazocin for 1 month followed by 4 months of PO Clindamycin. His range of motion improved to his premorbid state after rotator cuff repair when reviewed in the follow-up clinic 3 months after his rotator cuff surgery.

Presentation of deep infections following surgery may be subtle and often result in a delay in diagnosis [1]. Patients commonly present with pain and restricted range of motion. Most patients are afebrile and have normal white blood cells but may have elevated inflammatory markers [1, 2]. Findings of osteomyelitis on plain radiograph are subtle and, therefore, can be easily missed by clinicians and may take up to 2 weeks to become evident [3, 4]. MRI is the gold standard for detection and evaluation of osteomyelitis, and may detect changes as early as 3 to 5 days [4]. 

Medical conditions such as diabetes mellitus, decubitus ulcers, and trauma injuries such as open fracture, intravenous drug use, or venous insufficiency are common risk factors for developing osteomyelitis [5]. The patient discussed in this case had no known risk factors. 

Literature review found limited studies on deep infections after rotator cuff repair. Rate of chronic infection (including osteomyelitis and septic arthritis) after open rotator cuff surgery varies from 0.27% to 1.7% [6]. Treating deep infection after rotator cuff repair is possible but if missed, substantial functional limitations are common and debilitating [7]. Propionibacterium had been identified as the most common organism in deep infection after rotator cuff repair with an incident rate of almost 51% [7]. This coincides with culture from biopsy specimens of this patient and together with the presentation, lead, us to hypothesise that this patient had a chronic, low-grade deep infection.

The relationship between suture material and infection after rotator cuff repair was investigated by Boehm et al. [8], who compared Ethibond to polydioxanone (PDS) suture in a prospective randomised study on rotator cuff repair. There was no significant difference found between absorbable and non-absorbable suture in postrepair infection rate, at 24–30-month followup. Multifilament braided suture (i.e., Ethibond) has higher surface area for bacterial adhesion and therefore found to have higher rate of infection compared to monofilament suture [6, 9]. In regards to mechanical strength, multifilament suture is superior compared to monofilaments suture, therefore more commonly used in rotator cuff repair [5]. However braided and absorbable suture materials demonstrate similar mechanical properties to braided polyester such as Ethibond. When deep infections occur, suture and suture anchors usually require removal to treat the infection. Mirzayan et al. [6] retrospectively reviewed patients presenting with deep infection after rotator cuff repair surgery. They found that all non-absorbable sutures required removal to adequately treat the infection. In our case, the Ethibond suture was removed during open washout surgery along with debridement of the surrounding chronic granulation tissue. 

This case report raises the question of selecting multifilament absorbable suture for rotator cuff repairs, with similar mechanical properties to non-absorbable suture, and the potential benefit of reduced incidence of chronic deep infection.

In conclusion, initial presentation of deep infection can be subtle and easily missed by clinicians as presented in this case report. Deep infection after rotator cuff surgery is rare but treatable and a delay in diagnosis can lead to debilitating complications. Surgeons should consider the possibility of low-grade deep infection in patients presenting with shoulder pain after rotator cuff surgery.

## Figures and Tables

**Figure 1:**
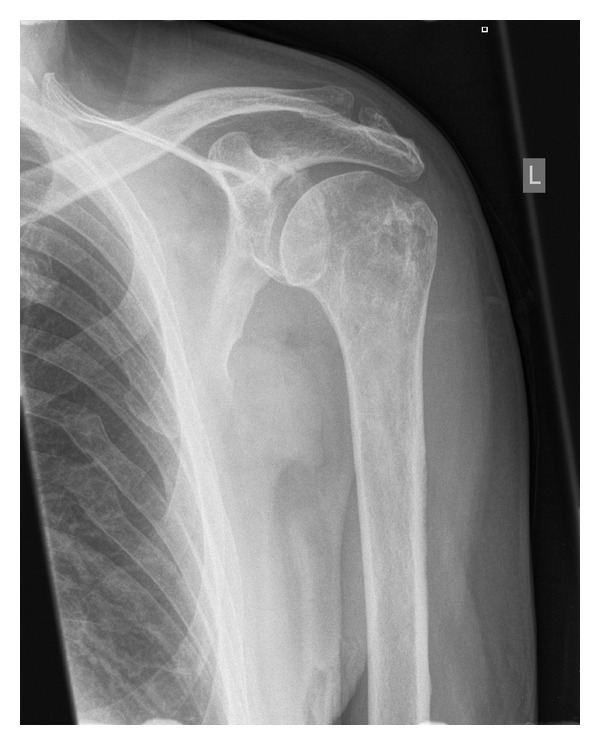
X ray left shoulder.

**Figure 2:**
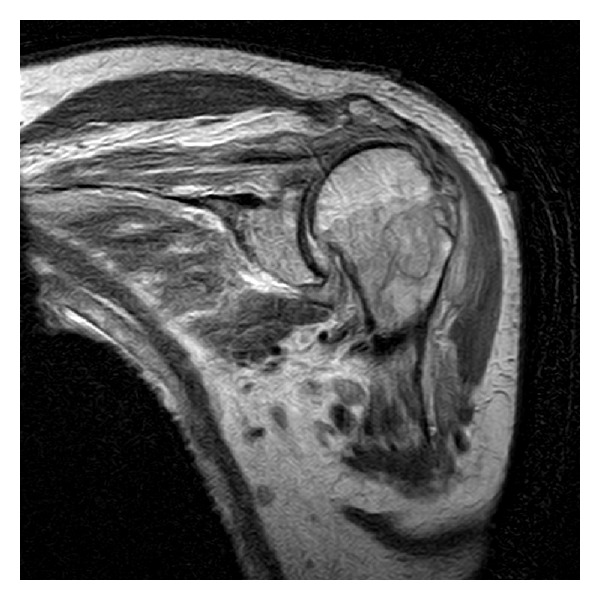
MRI left shoulder.

**Figure 3:**
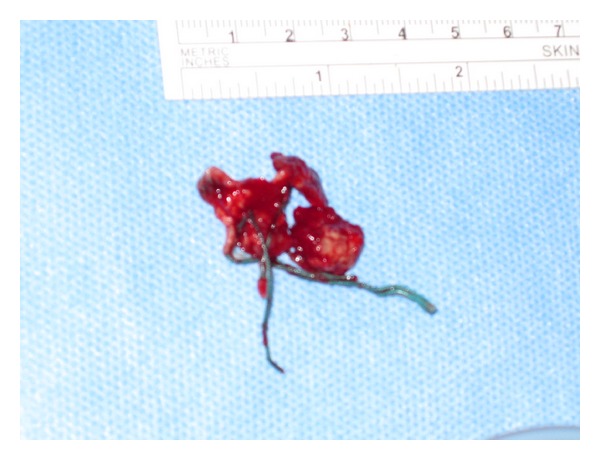
Excised Ethibond suture as focus of infection.
